# 2803. The Medical Trends and Economic Burden of XDR Typhoid Compared to Non-XDR Typhoid: A Single Center Retrospective Study from Karachi, Pakistan

**DOI:** 10.1093/ofid/ofad500.2414

**Published:** 2023-11-27

**Authors:** Muneeba Ahsan Sayeed

**Affiliations:** Sindh Infectious Diseases Hospital & Research Center/ Dow University of Health Sciences, Karachi, Sindh, Pakistan

## Abstract

**Background:**

In 2016, Pakistan reported the first outbreak of an Extensively Drug Resistant (XDR) typhoid showing susceptibility only to Meropenem and Azithromycin which created a treatment dilemma and raised concerns not only in developing countries but also globally. In this study, we compared the medical trends and economic burden of XDR typhoid with non-XDR typhoid at a tertiary care hospital of Karachi, Pakistan.

**Methods:**

A retrospective study was conducted at the Sindh Infectious Diseases Hospital & Research Centre Karachi, Pakistan from June 2022 to April 2023. All hospitalized patients 14 years and above with confirmed Salmonella *typhi* on blood culture were included. Medical records were reviewed for clinical data and antibiotic cost. Statistical analysis was done using SPSS.

**Results:**

Thirty-five patients were included of which 23 (65.7%) had XDR typhoid while 12 (34.3%) patients had non-XDR typhoid. Of the 12 non-XDR typhoid cases, nine were MDR (Multi Drug Resistant) while three had ampicillin sensitive strain. The mean age was 21.14 years with 27 (77.1%) males. Compared to non-XDR, majority of the XDR typhoid patients had diarrhea (60.9% vs. 16.7%, p-value= 0.02, OR 7.7, 95% CI: 1.3-44.0). XDR typhoid was treated with either Meropenem alone or Meropenem and Azithromycin combination while MDR typhoid was treated with Ceftriaxone. The time to defervescence was prolonged in XDR typhoid compared to non-XDR typhoid (7.95 days vs. 4.67 days, p-value= 0.007, OR 1.6, 95% CI: 1.14-2.3), with higher per day cost of antibiotics and total inpatient-antibiotic cost (US$20.3 vs. US$11, p-value= 0.006, OR 1.4, 95%CI 1.1-1.9) and (US$193 vs. US$139, p-value= 0.048] respectively. Within XDR group, the mean defervescence time was more in patients who received combined Meropenem and Azithromycin compared to Meropenem alone group (10.1 days vs 6.4 days, p-value=< 0.001) with a consequent higher per day in-patient antibiotic cost (USD$22 vs USD$ 18.8, p-value= < 0.001) and total in-patient cost (USD$ 235 vs. USD$ 158K, p-value= 0.009).

**Conclusion:**

XDR typhoid has emerged not only as a heath care but also as an economic challenge owing to its prolonged defervescence time and increased per day/ total in-patient antibiotic cost.

**Disclosures:**

**All Authors**: No reported disclosures

